# TMEM206 gene knockout improves balance performance in SCA1 transgenic mice

**DOI:** 10.1016/j.ibneur.2025.11.012

**Published:** 2025-11-20

**Authors:** Jia-Hui Zhang, Yu Qin, Shun-Chang Sun

**Affiliations:** a Department of Laboratory Medicine, Xinrui Hospital, Xinwu District, Wuxi 214112, China; b Department of Laboratory Medicine, Ruijin Hospital, Shanghai Jiao Tong University School of Medicine, Shanghai 201801, China

**Keywords:** TMEM206, Spinocerebellar ataxia type 1, Ataxin-1, Knockout, Motor coordination

## Abstract

TMEM206 was identified as a conserved chloride channel that underlies widely expressed, proton-activated, outwardly rectifying chloride currents. Spinocerebellar ataxia type 1 (SCA1) is one of polyglutamine diseases, and is characterized as a progressive and autosomal dominant genetic disease, which is caused by an increasing number of CAG repeats in the ataxin-1 gene. TMEM206 was confirmed to interact with ataxin-1. This study suggests that TMEM206-ataxin-1 interaction might involve in the pathological mechanisms of SCA1. To elucidate the mechanisms of SCA1 involved in proton-activated chloride channel gating, we bred TMEM206 knockout mice using SCA1 model mice. Motor coordination and balance in mice was evaluated using rotarod test and grip strength. These studies showed that genetic depletion of TMEM206 has slight impacts on the SCA1 mice weight, and partially improves motor incoordination in Atxn1^154Q/2Q^ mice. No alteration in grip strength was found in Atxn1^154Q/2Q^ mice with depletion of the TMEM206 gene. Our studies indicate that the TMEM206 knockout appears to emerge as a potential therapy method for SCA1 mice.

## 1 Introduction

Transmembrane protein 206 (TMEM206) was recently identified as a proton-activated chloride (PAC) channel protein, which plays an important role in neuronal death (Liu et al., 2025). TMEM206 shows a ubiquitous expression across different tissues, its role in physiological functions and pathologies have not been fully understood (Koylass et al., 2025). TMEM206 has been found to involve in acid-induced cell death in human cells. Additionally, TMEM206 has been shown to regulate the shrinkage and acidification of macropinosomes, and prevent endosomal hyperacidification (Osei-Owusu et al., 2022). A recent study by us revealed TMEM206 may be associated with chloride ion transport across corneal epithelium or endothelium in mice (Yang et al., 2024). Spinocerebellar ataxia type 1 (SCA1) is a fatal, dominantly inherited neurodegenerative disorder caused by CAG trinucleotide expansion in exon 8 of the ATXN1 gene which causes the formation of an expanded polyglutamine (polyQ) repeat within the ataxin-1 protein, leading to the premature degeneration of cerebellar Purkinje cells (Sheeler et al., 2024). A previous study showed that ataxin-1 interacted with TMEM206 in HEK-293T cells, however, mutant ataxin-1 lost protein interaction with TMEM206 (Chen et al., 2022). We speculated that ataxin-1-TMEM206 interaction might be contribute to the PAC channel gating. To elucidate the possible molecular mechanisms of SCA1 involved in PAC channel gating, we developed TMEM206 knockout mice using SCA1 model mice encoding ATXN1^154Q/2Q^. In this study, we found that TMEM206 gene knockout can improve balance performance in SCA1 model mice.

## 2 Materials and methods

### 2.1 Mice

Mice carrying the Atxn1^154Q/2Q^ mutation on the C57BL/6 J strain were obtained from The Jackson Laboratory (Bar Harbor, Maine) and subsequently bred at Gempharmatech Co., Ltd (Nanjing, China). TMEM206 knockout mice (TMEM206^+/-^) were generated by Gempharmatech Co., Ltd (Nanjing, China) using CRISPR-Cas9 gene editing technology with mice in the C57BL/6 J background. TMEM206 knockout mutation was confirmed using PCR technology and Sanger sequencing on the genomic DNA extracted from the mouse tail biopsies. Atxn1^154Q/2Q^ mice were bred to TMEM206^+/-^ mice, then, the F1 TMEM206^+/-^/Atxn1^154Q/2Q^ mice were backcrossed to TMEM206^+/-^/Atxn1^154Q/2Q^ to get the F2 TMEM206^-/-^/Atxn1^154Q/2Q^ mice. Mice were housed under natural environmental conditions with a 12 h light/dark cycle with dim light (50 lx) during the day. All animal experiments were approved by the Animal Ethics Committee of Wuxi Xinwu District Xinrui Hospital (No. SHAP20240913–1). All mice experiments were carried out in accordance with the U.S. National Institutes of Health guidelines and Animal Research: Reporting in Vivo Experiments guidelines. Sixteen male mice including six Atxn1^154Q/2Q^ mice, five TMEM206^-/-^/Atxn1^154Q/2Q^ mice and five wild-type littermate controls were used for rotarod test and grip strength. Body weight of mice was measured at 5, 10, 20, 30 and 40 weeks.

### 2.2 Rotarod test

Motor coordination and balance in mice was evaluated using Rotarod test, mice underwent 3 days of training on a Rotarod apparatus (XinRuan XR-6D, Shanghai) at a constant rotational speed of 20 rpm. Each of the training sessions lasted 5 min. On the test day, mice were tested at a Rotarod accelerating mode from 4 rpm to 40 rpm over 5 min for three trials with a 15-min rest interval between each trial. The latency to fall from the Rotarod was recorded for each trial, and the average time for latency to fall was computed for statistical analysis.

### 2.3 Grip strength

Grip strength was assessed with a computerized grip strength meter (XinRuan XR-501, Shanghai) at 5, 10, 20, 30 and 40 weeks for mice with established protocols. The grip strength meter consisted of a metal bar connected to a force transducer. Briefly, the protocols are as follows. To evaluate grip strength in the hindlimbs of the mice, the apparatus held the mouse by the tail root, allowing the mouse to grasp the metal bar with its hindpaws. The mice were first permitted to grasp a wire net cylinder with their forepaws in order to stop mice from gripping the metal bar with their forepaws while recording. The mouse was pulled backward by the tail until grip was lost if the mouse grasped the transducer metal bar with its hindpaws. The force of each test was recorded in grams by the device. The grip strength was measured in triplicate in each mouse.

### 2.4 Statistical analysis

Statistical analysis was performed according to the experimental design. Experimental data are expressed as mean ± standard deviation. Multigroup comparisons used one or two-way ANOVAs, whereas simple comparisons used Student’s two-tailed t test. Data were analyzed using GraphPad Prism 10.0 (GraphPad) and Excel 2020 (Microsoft). In each case, ns, *, **, and *** was used to indicate *p* > 0.05, *p* < 0.05, *p* < 0.01, and *p* < 0.001, respectively. *p* < 0.05 was considered statistically significant.

## 3 Results

### 3.1 Genetic depletion of TMEM206 has an impact on the SCA1 mice weight

**Fig. 1:**
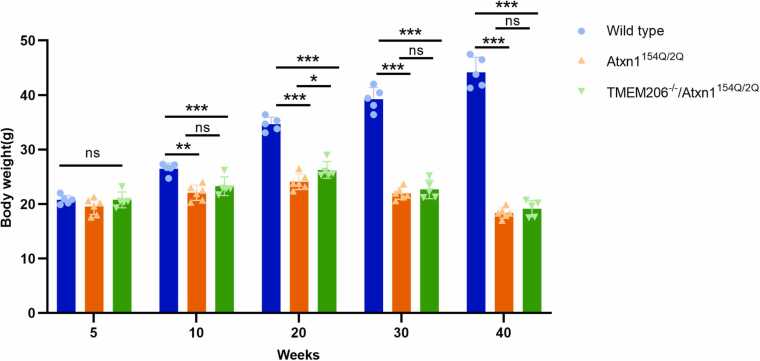
ANOVA revealed influence of the depletion of the TMEM206 gene has an impact on the Atxn1^154Q/2Q^ mice weight from 10 to 40 weeks of age.

To explore the functional consequences of the ATXN1/TMEM206 interaction, genetic depletion of the TMEM206 gene was obtained by inserting a stop codon in exon 2 of this gene by CRISPR-Cas9-mediated gene editing. As reported previously, Atxn1^154Q/2Q^ mice fail to gain weight after 8 weeks of age compared to wild-type littermate controls. We monitored the weight of mice over a 40-week period. No changes were observed in body weight in TMEM206^-/-^/Atxn1^154Q/2Q^ mice, Atxn1^154Q/2Q^ mice and wild-type littermates at five weeks of age (*p* > 0.05). Atxn1^154Q/2Q^ mice and TMEM206^-/-^/Atxn1^154Q/2Q^ mice showed significant weight reduction compared with wild-type mice starting from ten weeks of age (*p* < 0.001) (Fig. 1). We also detected a statistically significant amelioration of the TMEM206^-/-^/Atxn1^154Q/2Q^ mice weight loss compared with Atxn1^154Q/2Q^ mice at 20 weeks of age (*p* < 0.05), however, the amelioration of weight loss was observed but not statistically significant at 10, 30, and 40 weeks of age (*p* > 0.05) (Fig. 1). These studies indicate that depletion of the TMEM206 gene slightly improves the weight of Atxn1^154Q/2Q^ mice.

### 3.2 Depletion of TMEM206 partially improves motor incoordination in Atxn1^154Q/2Q^ mice

**Fig. 2:**
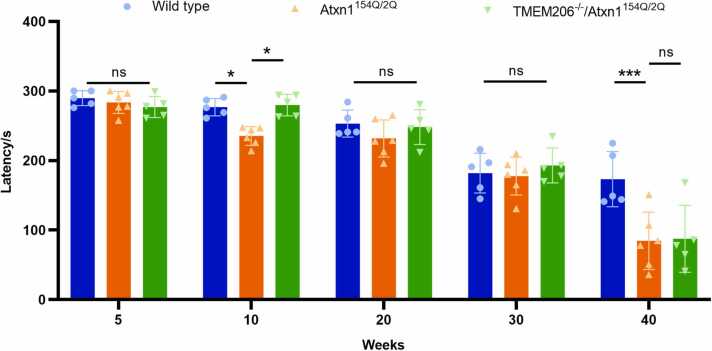
The depletion of TMEM206 improves the performance on the accelerating Rotarod of the Atxn1^154Q/2Q^ mice.

Previous studies showed that a decline in gross motor coordination is observed with increasing age. Atxn1^154Q/2Q^ mice developed motor incoordination at 10 weeks of age (*p* < 0.05). We showed that genetic depletion of the TMEM206 gene in Atxn1^154Q/2Q^ mice ameliorated motor coordination at 10 weeks of age compared with Atxn1^154Q/2Q^ mice without the depletion of the TMEM206 gene (*p* < 0.05), however, there was no statistical difference between the performance on the accelerating rotarod of the TMEM206^-/-^/Atxn1^154Q/2Q^ mice and the wild-type mice at 10 weeks of age (*p* > 0.05) (Fig. 2). In order to evaluate if the depletion of the TMEM206 gene in Atxn1^154Q/2Q^ mice is effective after symptom onset, we assessed the performance on the accelerating rotarod of Atxn1^154Q/2Q^, TMEM206^-/-^/Atxn1^154Q/2Q^ and wild-type mice after 10 weeks of age. It is interesting to find that the depletion of the TMEM206 gene in Atxn1^154Q/2Q^ mice seemed to improve performance in Atxn1^154Q/2Q^ mice at 20, 30, and 40 weeks of age, but their difference was not statistically significant (Fig. 2). These results suggest that the depletion of the TMEM206 gene can improve partially motor incoordination in Atxn1^154Q/2Q^ mice.

### 3.3 No alteration in grip strength in Atxn1^154Q/2Q^ mice with depletion of the TMEM206 gene

**Fig. 3:**
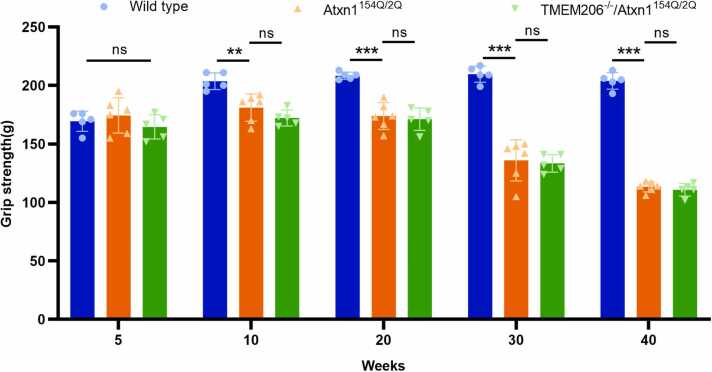
The depletion of TMEM206 does not rescue the grip strength impairment in Atxn1^154Q/2Q^ mice.

Grip strength was used to evaluate muscular function with three measurements made every other day. At 5 weeks of age, no differences in grip strength were observed between Atxn1^154Q/2Q^, TMEM206^-/-^/Atxn1^154Q/2Q^ and wild-type mice. Atxn1^154Q/2Q^ and TMEM206^-/-^/Atxn1^154Q/2Q^ mice developed a significant impairment in grip strength compared to wild-type mice from 10 to 40 weeks of age (*p* < 0.01, 0.001, 0.001, and 0.001, respectively). No alterations in grip strength were found between Atxn1^154Q/2Q^ and TMEM206^-/-^/Atxn1^154Q/2Q^ mice from 10 to 40 weeks of age (*p* > 0.05). The depletion of the TMEM206 gene did not rescue the grip strength impairment in Atxn1^154Q/2Q^ mice (Fig. 3).

## 4 Discussion

SCA1 is an autosomal dominant neurodegenerative disease caused by the abnormal expansion of a CAG repeat in the ATXN1 gene (Selimovic et al., 2025). The pathological mechanism of SCA1 is toxic gain of function by the mutant Atxn1 (Tejwani et al., 2024). Currently, there is no disease-modifying treatment available for SCA1. SCA1 therapeutics has been tested in some disease model systems including animal models and cell culture (Lee et al., 2022). The Atxn1^154Q/2Q^ mice expressing mutant Atxn1 with 154 CAG repeats throughout the brain and spinal cord are the most frequently used SCA1 mouse models. The Atxn1^154Q/2Q^ mice show motor incoordination, muscle wasting, premature lethality, kyphosis, and cognitive deficits (Shorrock et al., 2024). TMEM206 has been identified as an acid-sensitive outwardly rectifying chloride ion channel (Mihaljević et al., (2023)). Recently, we found that TMEM206 interacted with ataxin-1, the SCA1 gene product (Chen et al., 2022). We speculate that the pathological mechanism of SCA1 may be associated with ataxin-1-TMEM206 interaction. In support of this hypothesis, we explored motor coordination and grip strength in TMEM206^-/-^/Atxn1^154Q/2Q^ mice.

TMEM206^-/-^ mice were appeared mostly normal and viable, indicating that TMEM206 is not required for mouse viability (Yang et al., 2019). Our previous study found that TMEM206^−/−^ mice were mostly normal, except that corneal edema was observed in four out of the 18 TMEM206^−/−^ mice (Yang et al., 2024). However, corneal edema was not observed in the 5 TMEM206^-/-^/Atxn1^154Q/2Q^ mice in this study. In this study, we focused on the depletion of the TMEM206 gene on body weight development in Atxn1^154Q/2Q^ mice. Our studies show that a significant amelioration of the Atxn1^154Q/2Q^ mice weight loss at 20 weeks of age when the TMEM206 gene is stably knocked out by CRISPR-Cas9 gene editing technology, however, differences are also observed but not statistically significant at 10, 30, and 40 weeks. Our results indicate the depletion of the TMEM206 gene increases slightly body weight gain in Atxn1^154Q/2Q^ mice. Future studies will investigate how TMEM206 plays a role in body weight gain in Atxn1^154Q/2Q^ mice.

The rotarod test is usually used to measure the motor coordination of mice, and is particularly sensitive in testing cerebellar dysfunction (Rosa et al., 2023). Using the rotarod test, we found the depletion of the TMEM206 gene ameliorates impairments in motor coordination in SCA1 mice at 10 weeks of age, and the TMEM206 depletion restores the motor coordination of the SCA1 mice to the level similar to that of wild-type littermate mice. Theoretically, the CRISPR/Cas9 gene editing technology can knockout the TMEM206 gene due to its potential for permanent exon skipping, which can abolish the PAC channel in cerebellar Purkinje cells in SCA1 mice. This result suggests that the depletion of the TMEM206 gene might become a therapy approach for SCA1 in the future. The PAC channel composed of the TMEM206 protein mediates chloride influx in neurons (Kang and Lee, 2024). We speculate that PAC channel is blocked by a conjugated molecule with TMEM206 and ataxin-1 based on our previous studies, the pathological mechanism of SCA1 may be associated with the open PAC channel mediated by the abnormally expanded polyglutamine tract in ataxin-1 with TMEM206, which may lead to chlorine influx-induced neuron death. Therefore, the depletion of the TMEM206 gene blocks the open PAC channel mediated the mutant ataxin-1. It is unexpected that the depletion of the TMEM206 gene seems to improve impairments in motor coordination in SCA1 mice after 20 weeks of age, but the performance was not significant. There is a possible explanation for the phenomenon seen in motor coordination in SCA1 mice with and without TMEM206 knockout at the different age. SCA1 is characterized by the presence of the mutant ataxin-1 aggregates, the aggregates are found in postmortem SCA1 patient cerebellar tissues and in Atxn1^154Q/2Q^ mice at later stages of disease (Maharjan et al., 2025). At early stages of disease, the mutant ataxin-1 do not form aggregates in Atxn1^154Q/2Q^ mice cerebellar tissues. The TMEM206 knockout remarkably ameliorates impairments in motor coordination in SCA1 mice at early stages. However, expansion of the polyQ tract in the mutant ataxin-1 protein results in the formation of aggregates at later stages of disease. Aggregation of the mutant ataxin-1 lead to disturbance of some cellular processes such as transcription, autophagy, axonal transport in SCA1 mice when they get older even if the TMEM206 gene has been knocked out (Tammara and Das, 2023). So, the depletion of the TMEM206 gene improves only partially motor incoordination in Atxn1^154Q/2Q^ mice.

Atxn1^154Q/2Q^ mice by introducing a 154-Q into exon 8 of the ATXN1 gene display more of the human SCA1 disease features such as motor incoordination, cognitive impairment, muscle wasting and premature death (Shorrock et al., 2024). Atxn1^154Q/2Q^ mice onset begins usually at 7–8 weeks and die about at 45 weeks of age while the life span of wild-type C57BL/6 J mice is 80–105 weeks (Wagner et al., 2016). The age of 40 weeks is near the end of life for Atxn1^154Q/2Q^ mice. Therefore, the latency is significantly lower in Atxn1^154Q/2Q^ mice than in wild-type mice at 40 weeks, but not 20 or 30 weeks of age.

Grip strength test was widely used to measure skeletal muscle strength and neurobehavioral deficits in mice (Bushart et al., 2020). SCA1 mice were identified to have motor neuron involvement (Tezenas du Montcel, et al., 2023). In this study, no impairments in grip strength were found in Atxn1^154Q/2Q^ and TMEM206^-/-^/Atxn1^154Q/2Q^ mice when compared to wild-type mice at 5 weeks of age. Atxn1^154Q/2Q^ and TMEM206^-/-^/Atxn1^154Q/2Q^ mice displayed significantly impaired neurobehavioral deficits on the grip strength assay when compared to wild-type mice after 10 weeks. Meanwhile, there were no significant differences in grip strength between Atxn1^154Q/2Q^ and TMEM206^-/-^/Atxn1^154Q/2Q^ mice. These results suggest that the TMEM206 depletion doesn’t alleviate skeletal muscle strength in SCA1 mice.

## CRediT authorship contribution statement

**Jia-Hui Zhang:** Resources, Methodology. **Shun-Chang Sun:** Writing – review & editing, Writing – original draft, Conceptualization. **Yu Qin:** Formal analysis, Data curation.

## Funding

This study was supported by grants from Wuxi Health Commission Project (No. M202301).

## Conflicts of Interest

The authors declare that they have no known competing financial interests or personal relationships that could have appeared to influence the work reported in this paper.
